# Malignant Salivary Gland Tumors in a Tertiary University Hospital in Northern Spain

**DOI:** 10.3390/jcm14010046

**Published:** 2024-12-25

**Authors:** Carlos M. Chiesa-Estomba, Alfonso Rodriguez-Urzay, Maria Landa-Garmendia, Ekhiñe Larruscain-Sarasola, Jose A. González-García, Jon A. Sistiaga-Suarez, Leyre González-Vallejo, Carlos Blanco-García

**Affiliations:** 1 Department of Otorhinolaryngology, Biodonostia Research Institute, Osakidetza, Donostia University Hospital, 20014 San Sebastian, Spain; 2 Radiation Oncology Department, Osakidetza, Donostia University Hospital, 20014 San Sebastian, Spain

**Keywords:** salivary glands, malignant, parotid, submandibular

## Abstract

(1) **Background**: Salivary gland tumors (SGTs) are a rare and diverse group of neoplasms arising in the parotid, submandibular, sublingual, and minor salivary glands distributed throughout the upper aerodigestive tract. Given the rarity and complexity of MSGTs, understanding their epidemiology across diverse populations is crucial for improving diagnostic and therapeutic strategies. (2) **Methods**: A retrospective analysis involving 45 patients diagnosed with malignant salivary gland tumors and treated with curative intention between 1 July 2016 and 1 July 2021 in a tertiary academic hospital was performed. (3) **Results**: Histologically, the most common subtype was adenoid cystic carcinoma in 12 (26.7%) cases, followed by carcinoma ex-pleomorphic adenoma in 7 (15.6%) cases, mucoepidermoid carcinoma in 6 (13.3%) cases, and adenocarcinoma in 6 (13.3%) cases. The majority of tumors were located in the parotid gland in 28 cases (62.2%). The three-year overall survival rate was 80% and the three-year specific survival rate was 86.7%. Tumor grade was significantly associated with local recurrence and the appearance of distant metastasis during the follow-up period (*p* = 0.04). We also evidenced a worse survival in patients with advanced T-Stage (*p* = 0.038) and positive nodes in the neck (*p* = 0.015). (4) **Conclusions**: Overall, our study reinforces the prognostic significance of tumor grade, T-Stage and N-Stage while challenging the conventional understanding of factors such as PNI, LNM, and margin status.

## 1. Introduction

Salivary gland tumors (SGTs) are a rare and diverse group of neoplasms arising in the parotid, submandibular, sublingual, and minor salivary glands distributed throughout the upper aerodigestive tract. These tumors present a wide spectrum of histological subtypes, making their diagnosis and management particularly challenging. The World Health Organization (WHO) recognizes over 20 distinct histological subtypes of SGTs, with malignant variants including mucoepidermoid carcinoma (MEC), adenoid cystic carcinoma (AdCC), acinic cell carcinoma (ACC), carcinoma ex pleomorphic adenoma (CExPA), salivary duct carcinoma (SDC), and adenocarcinoma not otherwise specified (NOS) being among the most common [1].

Globally, malignant salivary gland tumors (MSGTs) are uncommon, accounting for approximately 0.3% of all malignancies with an incidence between 0.7 and 0.9/100,000 habitants [2,3,4]. Their rarity, combined with diverse histology and clinical presentations, makes it difficult to establish diagnosis and treatment protocols. Epidemiological data from various regions reveal significant differences in incidence rates and histological distributions. In Shanghai, China, the incidence of MSGTs was reported as 7.99 per million people, with a male-to-female ratio of 1.1:1 and the parotid gland being the most commonly affected site [2,5]. Similarly, in Iceland, a nationwide study spanning 30 years identified an incidence of 0.59 per 100,000 among men and 0.79 per 100,000 among women, with mucoepidermoid carcinoma, acinic cell carcinoma, and adenoid cystic carcinoma being the predominant histological subtypes [6].

The etiology of MSGTs remains poorly understood. Radiation exposure is one of the most established risk factors, particularly in patients who received radiation therapy during childhood [6]. Unlike other head and neck cancers, the association between tobacco and alcohol use and MSGTs is less clear. Additionally, autoimmune conditions such as Sjögren’s syndrome have been linked to the development of salivary gland lymphomas, particularly mucosa-associated lymphoid tissue (MALT) lymphomas [7]. Most recently, the increasing research in driver mutations and targeted therapies in MSGTs, like the over-expression of the androgen receptor (AR) and HER-2 amplification reported in SDCs, or the use of broad-spectrum tyrosine kinase inhibitor (TKI) as VEGFR blocker help doctors and researchers to better understand the genetic landscape in this subset of malignancies [8].

Given the rarity and complexity of MSGTs, understanding their epidemiology across diverse populations is crucial for improving diagnostic and therapeutic strategies. This study aims to evaluate the demographic, clinical, and histopathological characteristics of patients diagnosed with malignant salivary gland tumors in a tertiary university hospital in northern Spain, looking to contribute to the broader knowledge of this disease.

## 2. Materials and Methods

A retrospective analysis involving 45 patients diagnosed with malignant salivary gland tumors and treated with curative intention between 1 July 2016 and 1 July 2021 in a tertiary academic hospital was performed (Ethical code number: CCH-230123—11 September 2023). The inclusion criteria consisted of patients diagnosed with malignant minor and major salivary gland tumors of varying histological subtypes and anatomical locations, with a minimum follow-up period of 6 months after surgery and a maximum of 36 months for survival analysis. Patients with incomplete clinical records, skin metastasis or those lost to follow-up were excluded.

Demographic, clinical, histopathological, and treatment data were extracted from medical records. Variables collected included age, sex, smoking and alcohol habits, comorbidities like high blood pressure (HBP) and diabetes mellitus, tumor histology, anatomical location, tumor staging (T, N, M stages), tumor differentiation, perineural invasion (PNI), lymphovascular invasion (LVI), surgical margins, and details of complementary treatments (radiotherapy, chemo-radiotherapy). Survival outcomes, including overall survival and disease-specific survival, were recorded. All cases were discussed in the multidisciplinary tumor board (MDT) before and after surgery.

Histological classification of salivary gland tumors followed the 4th edition of the *WHO Blue Book Classification* [9]. Tumor staging was determined based on the TNM staging system by the American Joint Committee on Cancer (AJCC) [8th edition], categorizing tumors into T1, T2, T3, and T4a stages. Nodal involvement was classified as N0, N1, N2a, and N3b. M0 classification was confirmed for all patients, indicating no distant metastasis at the time of diagnosis.

Statistical analysis was conducted with SPSS for Macintosh Version 21.0 (IBM Corp, Armonk, NY, USA). Descriptive statistics were used to summarize patient demographics, tumor characteristics, and treatment modalities. Continuous variables were reported as mean ± standard deviation, while categorical variables were presented as frequencies and percentages. The normality of the distribution of continuous data was assessed using the Kolmogorov–Smirnov test. The relationship between nonparametric variables was studied using the chi-square test. Parametric variables were compared with the independent-sample *t* test. Survival analysis was performed using the Kaplan–Meier method and log-rank test, and graphs were drawn. Overall survival (OS) and disease-specific survival (DSS) rates were calculated for the cohort. OS was defined as the time between the date of surgery or the end of treatment and that of death due to every possible cause, DSS was defined as the time from the date of surgery or the end of treatment to the date of death related to the salivary gland malignancy. The follow-up period extended until 1 August 2024, with survival outcomes determined based on patient records and death certificates where applicable. A *p*-value of <0.05 was accepted as statistically significant. Variables that were considered time-to-event were entered into multivariate regression analysis performed using the Cox proportional hazards model and the stepwise method. Hazard ratios (HRs) with 95% confidence intervals (CI) were calculated.

This study was conducted in accordance with the principles outlined in the Declaration of Helsinki. Ethical approval was obtained from the Institutional Review Board (IRB number: CCH-012548). Given the retrospective nature of the study, a waiver of informed consent was granted by the IRB.

## 3. Results

During the five-year study period, a total of 282 primary salivary gland tumors were surgically managed in our department. Among these, the parotid gland was the most frequently affected gland, with 89% of the tumors (227 patients) being benign and 11% (28 patients) malignant. In the submandibular gland, there was a higher incidence of malignancy, with 43% of tumors (6 patients) identified as malignant and 57% (8 patients) as benign. Minor salivary tumors arising in other subsites of the aero-digestive tract, were less common but demonstrated the highest malignancy rate, with 84.6% (11 patients) being malignant and only 15.4% (2 patients) benign (Table 1).

A total of 45 patients were included in this study, with a mean age of 56 ± 15 years (range: 18–87 years). Among the patients, 26 (57.8%) were male and 19 (42.2%) were female. Regarding smoking status, 9 (20%) were active smokers, 26 (57.8%) were non-smokers, and 10 (22.2%) were former smokers. Alcohol consumption was reported by 15 (33.3%) patients. HBP was noted in 17 (37.8%) patients, and diabetes mellitus was present in 6 (13.3%) patients. The Kolmogorov–Smirnov *p*-value was 0.673, indicating that our age data were normally distributed. In all cases, surgery was established as the first treatment option. One patient with a Mucoepidermoid carcinoma with distant metastasis found during the diagnosis work-up was excluded from the final analysis (Table 2).

Histologically, the most common subtype was adenoid cystic carcinoma in 12 (26.7%) patients, followed by carcinoma ex-pleomorphic adenoma in 7 (15.6%) patients and mucoepidermoid carcinoma in 6 (13.3%) patients. Less frequent histological subtypes included myoepithelial carcinoma in 4 (8.9%) cases, acinar cell carcinoma in 4 (8.9%) patients, polymorphus adenocarcinoma and not otherwise specified (NOS) Adenocarcinoma in 3 (6.6%) patients each respectively, basal cell adenocarcinoma in 2 (4.4%) patients, salivary duct carcinoma in 1 (2.2%) patient, and small-cell neuroendocrine carcinoma in 1 (2.2%) patient, lymphoepithelioma in 1 (2.2%) patient, and small round cell desmoplastic tumor in 1 (2.2%) patient. Due to the high variability among histologies and the low-sample size, survival analysis among histological subtypes was not performed. Three-year local control was achieved in 88.8% of patients (Table 3).

In terms of anatomical distribution, the majority of tumors were located in the parotid gland in 28 patients (62.2%) and followed by the submandibular gland in 6 patients (13.3%). Tumors also occurred in other locations including the oropharynx in 3 patients (6.7%), oral cavity in 2 patients (4.4%), maxillary sinus in 2 patients (4.4%), nasal cavity in 2 patients (4.4%), hypopharynx in 1 patient (2.2%), and the larynx in 1 patient (2.2%) (Table 3).

Tumor staging revealed that 14 (31.1%) patients were T1 stage, 22 (48.9%) patients were T2, 4 (8.9%) patients were T3, and 5 (11.1%) patients were T4a. Regarding nodal involvement, 39 (86.7%) patients were N0, 2 (4.4%) patients were N1, 1 (2.2%) patient was N2a, and 3 (6.7%) patients were N3b. Histopathological differentiation revealed that 12 (26.7%) patients were grade 1, 9 (20%) patients were grade 2, and 24 (53.3%) patients were grade 3. Moreover, PNI was observed in 13 (28.9%) cases, while LVI was present in 2 (4.4%) cases (Table 1). Surgical margins were free of disease in 34 (75.6%) patients, while 11 (24.4%) patients had affected margins. Adjuvant postoperative treatment included radiotherapy in 24 (53.3%) patients and chemo-radiotherapy in 6 (13.3%) patients, while 15 (33.3%) patients did not receive any additional therapy. The main indications for adjuvat RT or CRT were positive margins, PNI and LVI.

**Figure 1:**
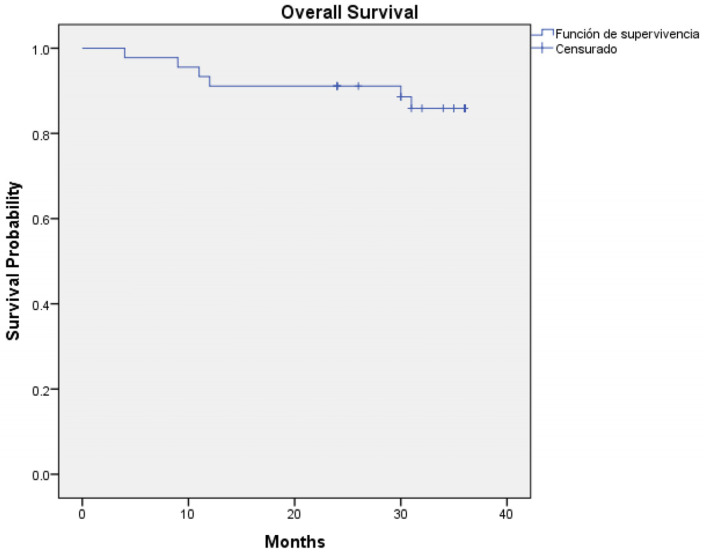
Kaplan–Meier regarding three-year overall survival (80%).

**Figure 2:**
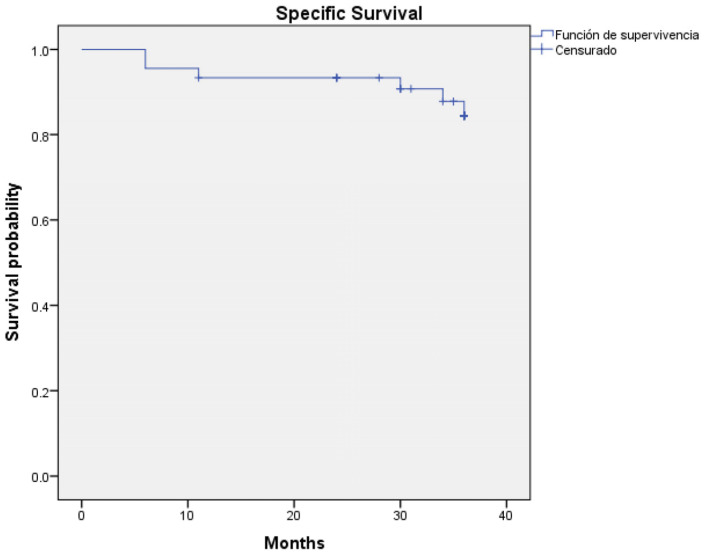
Kaplan–Meier regarding three-year specific survival (86.7%).

**Figure 3:**
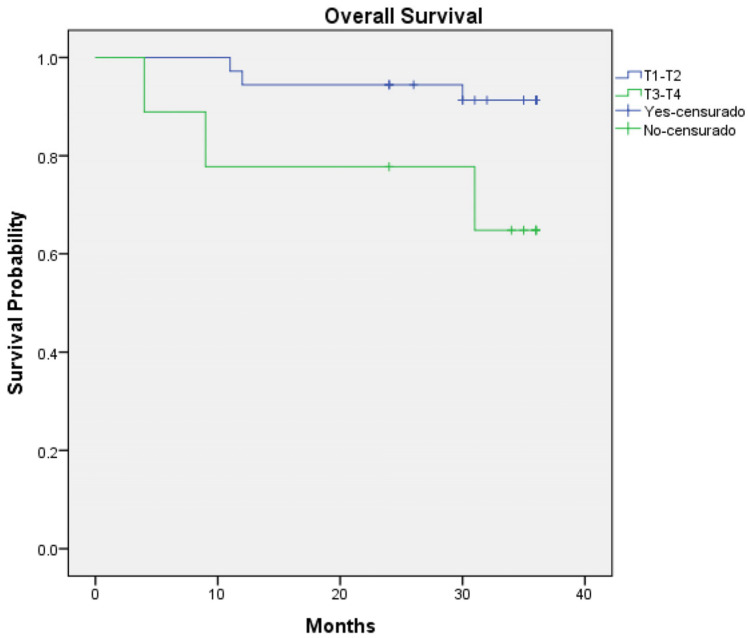
Kaplan–Meier regarding three-year overall survival according to T-Stage.

**Figure 4:**
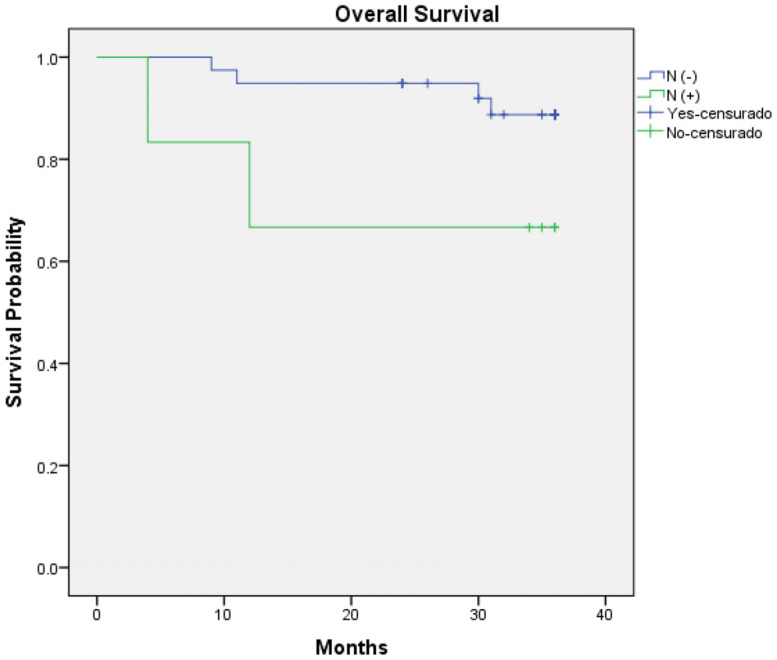
Kaplan–Meier regarding three-year overall survival according to N-Stage.

Regarding survival outcomes, the three-year overall survival rate was 80% (36 out of 45 patients—95% CI: 0.927 to 1.036) (Figure 1) and three-year disease-specific survival was 86.7% (39 out of 45 patients—95% CI: 0.944 to 1.045) (Figure 2). Log-rank analysis regarding T-Stage (T1–T2 vs. T3–T4) (Figure 3) and N-Stage (N-Positive vs. N-Negative) (Figure 4), demonstrated a worse survival in patients with advance T-Stage (*p* = 0.038, 95% CI: 1.094 to 4.801) and positive nodes in the neck (*p* = 0.015, 95% CI: 0.490 to 1.461).

The most frequently distant metastatic histology diagnosed during the follow-up period were high-grade AdCC (3 of 5 patients = 60%), polymorphus adenocarcinoma (1 of 5 patients = 20%), and small-cell neuroendocrine carcinoma (1 of 5 patients = 20%). The origin of DM was more commonly founded in those patients affected by major salivary gland tumors (4 of 5 cases = 80%) with a statistical significant difference (*p* = 0.048). (Table 3) And regarding the site affected, 4 patients were diagnosis with a Lung DM and 1 with a Liver DM (Table 4).

The univariate analysis of factors associated with local recurrence (LR) and distant metastasis (DM) revealed several key findings. Tumor grade was significantly associated with local recurrence and the appearance of distant metastasis during the follow-up period (*p* = 0.04). However, perineural invasion did not show a significant impact on outcomes, with no statistical difference regarding LR (*p* = 0.249) and the risk of DM (*p* = 0.641). Similarly, despite the fact that lymph node metastasis was more common in major salivary gland tumors, we did not find significant difference regarding the risk of regional metastasis (*p* = 0.171). Moreover, no significant differences were found between risk of LR and DM according to treatment modalities (RT vs. CRT) (*p* = 0.359), margin status (*p* = 0.344 and *p* = 0.806) or T-Stage (*p* = 0.263 and *p* = 0.235). (Table 5). Therefore, in the Cox Proportional multivariate analysis, just the T-Stage was associated with a worse survival (*p*: 0.018; HR: 5 (95% CI: −0.062 to 10.106) (Table 6).

The univariate analysis of factors associated with local recurrence (LR) and distant metastasis (DM) revealed several key findings. Tumor grade was significantly associated with local recurrence and the appearance of distant metastasis during the follow-up period (*p* = 0.04). However, perineural invasion did not show a significant impact on outcomes, with no statistical difference regarding LR (*p* = 0.249) and the risk of DM (*p* = 0.641). Similarly, despite the fact that lymph node metastasis was more common in major salivary gland tumors, we did not find significant difference regarding the risk of regional metastasis (*p* = 0.171). Moreover, no significant differences were found between risk of LR and DM according to treatment modalities (RT vs. CRT) (*p* = 0.359), margin status (*p* = 0.344 and *p* = 0.806) or T-Stage (*p* = 0.263 and *p* = 0.235) (Table 4). Therefore, in the Cox Proportional multivariate analysis, just the T-Stage was associated with a worse survival (*p*: 0.018; HR: 5 (95% CI: −0.062 to 10.106) (Table 5).

## 4. Discussion

With this study the authors tried to contribute to the growing body of evidence on prognostic factors and outcomes in patients with MSGTs. In our analysis, high-grade tumors were significantly associated with increased rates of local recurrence (LR) and distant metastasis (DM) (*p* = 0.04), consistent with prior studies demonstrating that higher-grade tumors are more likely to exhibit aggressive behavior and poorer prognosis [10,11]. as well as a critical determinant of survival in salivary gland malignancies [12,13]. Moreover, an advanced T-Stage, and the presence of positive neck nodes were associated with a poorer survival outcome.

The median age of the study population was 56 years, and a nearly balanced gender distribution was observed, which reflects the typical demographic distribution seen in salivary gland malignancies. Regarding histological subtypes, MEC is considered the most common histology in some studies [14,15]. The predominance of adenoid cystic carcinoma (AdCC) in our cohort, accounting for 26.7% of cases, aligns with existing literature, where AdCC is frequently reported as the most common subtype in both major and minor salivary gland tumors [16].

Despite this, we were not able the analyze survival outcomes according to histological subtypes. Findings across the literature support the heterogeneous behavior of salivary gland tumors, which are influenced not only by the histological subtype, but also by a wide range of factors, including anatomical location, tumor grade, and patient-specific characteristics [16,17,18]. The anatomical distribution of tumors in our study, predominantly affecting the parotid gland (62.2%), is also in line with established patterns observed in salivary gland carcinoma cohorts [16]. Therefore, tumor staging revealed that the majority of cases were early-stage (T1–T2), comprising 80% of the cohort. This distribution is encouraging as early-stage tumors are generally associated with better outcomes, particularly when treated surgically with curative intent.

The presence of nodal involvement in a small proportion of patients (13.3%), all of them affected by a parotid gland malignancy, aligns with the typically low but significant risk of regional spread in salivary gland malignancies. Our study did not find a significant difference between major and minor salivary gland tumors in terms of Lymph Node Metastasis (LNM) (*p* = 0.171). However, as in previous studies published by Spiro et al. and Vander Poorten et al., LNM was a critical predictor of survival in our cohort (*p* = 0.015) [19,20], highlighting the benefit of early diagnosis in these cases. By contrast, a recently published meta-analysis performed by Ho et al. regarding the incidence of level-specific cervical node metastasis in primary parotid malignancies, reported a significant rate of occult neck metastases in up to 32% of patients in the clinical negative neck and 89% in the clinically positive neck in other neck levels, recommending individuals an elective treatment of the neck through a selective neck dissection including levels II to III, with the inclusion of level IV based on clinical judgment in primary parotid malignancies [21]. Moreover, in another publication by Zhang et al. the authors found an overall rate of occult neck metastasis of 27% in parotid salivary gland malignancies, 35% in submaxilar and sublingual malignancies and 15% in other minor salivary gland malignancies, with the neck levels I-III being the most commonly affected [22]. In this vein, looking into our results, as well as the information available in the literature, discrepancies make it mandatory to emphasize the need for a tailored approach to the neck, individualizing the management according to the histology and clinical factors.

Perineural invasion has been widely recognized as a negative prognostic factor in head and neck cancers, including salivary gland tumors. However, in our study, PNI did not show a statistically significant association with LR (*p* = 0.249) or DM (*p* = 0.641). This finding contrasts with research by Wang et al. and Jaber, who reported that PNI is often associated with worse outcomes, particularly in adenoid cystic carcinoma [23,24]. The lack of significance in our cohort may reflect differences in tumor subtypes or the relatively small sample size as well as influence the short follow-up in AdCC patients, where recurrence usually occurs several years after initial treatment.

Surgical margin status is a well-established prognostic factor in many cancers, including salivary gland tumors. However, in our study, margin involvement was not significantly associated with a higher risk of LR (*p* = 0.344) or the appearance of DM (*p* = 0.806). This result diverges from previous studies that highlight the association of clear surgical margins with an improved local control and survival [11,16]. The inconsistency may be due to differences in surgical techniques, histopathological evaluations, the use of adjuvant therapy or sample sizes across studies. The role of adjuvant therapy, particularly the comparison between radiotherapy (RT) and chemoradiotherapy (CRT), remains contentious in the literature. In our cohort, no significant differences in outcomes were observed between patients treated with RT versus CRT (*p* = 0.359). This aligns with findings by Hay et al. and others, suggesting that the addition of chemotherapy to RT may not universally improve survival outcomes but could be beneficial in select cases based on tumor stage and histology [14].

In the present study, the three-year overall survival rate was 80%. The results are consistent with previous findings reported in the literature, like those published by Hay et al. reporting a five-year overall survival rate of 86%, suggesting a relatively stable survival outcome over a more extended period [14]. Similarly, Spiro et al. reported an overall survival rate of 75%, indicating consistency across different cohorts and methodologies [19]. Vander Poorten et al. also documented a survival rate of 68%, which is slightly lower but still comparable to the survival rates observed in our study [20]. Regarding disease-specific survival, our study found a three-year disease-specific survival rate of 86.7%, which is also comparable to previous findings, like those published by Allufi et al. which reported a disease-specific survival rate of 75%, showing some variation but still within a reasonable range in comparison with our study’s results [10]. Findings that support the robustness of current treatment approaches across different populations, offer a benchmark for future studies to evaluate novel therapies or interventions. However, we need to highlight the limitations of our analysis, due to the shorter follow-up in our cohort in comparison with the previous studies.

In our study, the most frequent metastatic histologic types during the follow-up period were high-grade AdCC (60%), adenocarcinoma (20%), and neuroendocrine carcinoma (20%). Results that are consistent with the literature were that higher-grade malignancies are the cause of more frequent distant metastases in these histologies [16]. The origin of DM was more commonly founded in the major salivary glands (80%), results regarding the most common affected site by DM showed that the lung was the main location with (80% of all metastatic patients) [25,26].

In recent years, several targetable molecular alterations have been identified in MSGCs, including HER2 upregulation, androgen receptor overexpression, Notch receptor activation, NTRK gene fusions, and RET alterations. Various clinical trials are underway exploring the efficacy of multikinase inhibitors, NTRK inhibitors, anti-HER-2 therapies, immune checkpoint inhibitors (PD-L1), and different combinations of these drugs. While some treatments have demonstrated significant improvements in patient outcomes, many studies are still in progress, which opens up multiple potential treatment alternatives in the near future [16,27].

Finally, we need to highlight the major limitations in our study, like the retrospective nature of the data analysis, the small sample size, and the lack of immunohistochemical and gen-profile analysis of the surgical specimens.

## 5. Conclusions

Overall, our study reinforces the prognostic significance of tumor grade, T-Stage, and N-Stage, while challenging the conventional understanding of factors such as PNI, LNM, and margin status. Given the complexity and rarity of salivary gland carcinomas, larger, multicenter studies with more extensive follow-up are necessary to clarify these associations and guide evidence-based management strategies. As emphasized in recent literature, individualized treatment planning, informed by comprehensive pathological and molecular profiling, remains essential for optimizing outcomes in these diverse and challenging malignancies.

## Figures and Tables

**Table 1:** Distribution regarding histology (Benign vs. Malignant) according to anatomical subsites.

Variable	Benign	%	Malignant	%
Parotid	227	89	28	11
Submandibular gland	8	57	6	43
Other locations	2	15.4	11	84.6

**Table 2:** Demographic variables of patients included.

Variables	N	%
Age	56 ± 15 (Min: 18/Max: 87)	
Sex		
Male	26	57.8
Female	19	42.2
Smoke		
Yes	9	20
No	26	57.8
Former	10	22.2
Alcohol		
Yes	15	33.3
No	30	66.7
HBP		
Yes	17	37.8
No	28	62.2
Diabetes mellitus		
Yes	6	13.3
No	39	86.7
T Stage		
T1	14	31.1
T2	22	48.9
T3	4	8.9
T4a	5	11.1
N Stage		
N0	39	86.7
N1	2	4.4
N2a	1	2.2
N3b	3	6.7
M0		
No	0	0
Complementary treatment		
No	15	33.3
Radiotherapy	24	53.3
Chemo-Radiotherapy	6	13.3
Anatomical location		
Parotid gland	28	62.2
Submandibular gland	6	13.3
Oropharynx	3	6.7
Oral Cavity	2	4.4
Maxillary Sinus	2	4.4
Nasal Cavity	2	4.4
Hypopharynx	1	2.2
Larynx	1	2.2

HBP = High Blood Pressure.

**Table 3:** Histological data.

Variables	N	%
Histology		
Adenoid Cystic Carcinoma	12	26.7
Carcinoma Ex-Pleomorfic Adenoma	7	15.6
Mucoepidermoid Carcinoma	6	13.3
Adenocarcinoma	6	13.3
Myoepitelial Carcinoma	4	8.9
Acinar Cell Carcinoma	4	8.9
Polymorphus adenocarcinoma	3	6.6
Non-Other Specified (NOS) Adenocarcinoma	3	6.6
Basal Cell adenocarcinoma	2	4.4
Salivary Duct Carcinoma	1	2.2
Small-cell Neuroendocrine Carcinoma	1	2.2
Linfoepitelioma	1	2.2
Small Round Cell Desmoplasic Tumor	1	2.2
Anatomical location		
Parotid gland	28	62.2
Submandibular gland	6	13.3
Oropharynx	3	6.7
Oral Cavity	2	4.4
Maxillary Sinus	2	4.4
Nasal Cavity	2	4.4
Hypopharynx	1	2.2
Larynx	1	2.2
Grade		
1	12	26.7
2	9	20
3	24	53.3
PNI		
Yes	13	28.9
No	32	71.1
LVI		
Yes	2	4.4
No	43	95.6
Margins		
Free	34	75.6
Affected	11	24.4

PNI = Perineural Invasion; LVI: lymphovascular invasion.

**Table 4:** Correlation between histological subtype and the type of recurrence (Local, regional, dstant).

Histology	Local	Lung	Liver
Adenocarcinoma	2	0	0
Adenoid Cystic Carcinoma	1	2	1
Myoepitelial Carcinoma	1	0	0
Carcinoma Ex-Pleomorfic Adenoma	4	0	0
Small-cell Neuroendocrine Carcinoma	0	1	0
Small Round Cell Desmoplasic Tumor	1	0	0
Mucoepidermoid Carcinoma	0	1	0

**Table 5:** Correlation between risk factors: lymph node metastasis (LNM), local recurrence (LR) and distant metastasis (DM). RT = Radiotherapy; CRT = Chemo-radiotherapy; PNI = Perineural invasion; LR = Local Recurrence; DM = Distant Metastasis; SG = Salivary gland.

Variable	N	*p*
Local recurrence and PNI Distant Metastasis and PNI	PNI (+) = LR: 4/PNI (−) = LR: 5 PNI (+) = DM: 1/PNI (−) = DM: 4	0.249 0.641
LNM—Major Versus Minor SG	LNM + Major SG: 6/34 LNM + Minor SG: 0/11	0.171
Local Recurrence/DM and Grade	Grade 1 and 2: LR: 2/DM: 0 (21) Grade 3: LR: 6/DM: 5 (24)	0.04
Local Recurrence or Distant Metastasis after RT or CRT	RT + = LR: 5/DM: 3 (24) CRT: LR: 3/DM: 1 (6)	0.359
Local Recurrence and Margin Status Distant Metastasis and Margin status	Margin (+) = LR: 3/Margin(−) = LR: 6 Margin (+) = DM: 1/Margin (−) =DM: 4	0.344 0.806
Recurrence risk and T-Stage Distant Metastasis and T-Stage	T1–T2 (6) vs. T3–T4 (3) T1–T2 (3) vs. T3–T4 (2)	0.263 0.235
Distant Metastasis and major or minor salivary gland malignancy	Major SG = 4/36 vs. Minor SG = 1/11	0.048

**Table 6:** Multivariate Cox Proportional Hazards regression model of OS and factors related to local control for the 45 patients with malignant salivary gland disease.

Variable	HR (95% CI)	*p*
T-Stage	5 (−0.062 to 10.106)	0.018
PNI	1.711 (−9.890 to 11.591)	0.871
Histological grade	2.267 (0.724 to 2.994)	0.112
Distant Metastasis	1.250 (0.358 to 4.355)	0.720

## Data Availability

Data are available under request.
